# Cu@Fe-Redox Capacitive-Based Metal–Organic Framework Film for a High-Performance Supercapacitor Electrode

**DOI:** 10.3390/nano13101587

**Published:** 2023-05-09

**Authors:** Supriya A. Patil, Pranav K. Katkar, Mosab Kaseem, Ghazanfar Nazir, Sang-Wha Lee, Harshada Patil, Honggyun Kim, Verjesh Kumar Magotra, Hoa Bui Thi, Hyunsik Im, Nabeen K. Shrestha

**Affiliations:** 1 Department of Nanotechnology & Advanced Materials Engineering, Sejong University, Seoul 05006, Republic of Koreagnazir@sejong.ac.kr (G.N.); 2 Department of Chemical and Biological Engineering, Gachon University, Seongnam-si 13120, Republic of Korea; pranavkatkar1793@gmail.com (P.K.K.); lswha@gachon.ac.kr (S.-W.L.); 3 Department of Electrical Engineering, Sejong University, Seoul 05006, Republic of Korea; harshadapatil.nanotech@gmail.com (H.P.); khgking11@naver.com (H.K.); 4 Quantum-Functional Semiconductor Research Center, Dongguk University, Seoul 04620, Republic of Korea; birju.srm@gmail.com; 5 Institute of Materials Science, Vietnam Academy of Science and Technology, Graduate University of Science and Technology, Hanoi 112400, Vietnam; hoabl30201811@gmail.com; 6 Division of Physics and Semiconductor Science, Dongguk University, Seoul 04620, Republic of Korea

**Keywords:** Cu-doped Fe-MOF, drop-cast film, bimetallic redox capacitive, supercapacitor

## Abstract

A metal–organic framework (MOF) is a highly porous material with abundant redox capacitive sites for intercalation/de-intercalation of charges and, hence, is considered promising for electrode materials in supercapacitors. In addition, dopants can introduce defects and alter the electronic structure of the MOF, which can affect its surface reactivity and electrochemical properties. Herein, we report a copper-doped iron-based MOF (Cu@Fe-MOF/NF) thin film obtained via a simple drop-cast route on a 3D-nickel foam (NF) substrate for the supercapacitor application. The as-deposited Cu@Fe-MOF/NF electrodes exhibit a unique micro-sized bipyramidal structure composited with nanoparticles, revealing a high specific capacitance of 420.54 F g^−1^ at 3 A g^−1^ which is twice compared to the nano-cuboidal Fe-MOF/NF (210 F g^−1^). Furthermore, the asymmetric solid-state (ASSSC) supercapacitor device, derived from the assembly of Cu@Fe-MOF/NFǁrGO/NF electrodes, demonstrates superior performance in terms of energy density (44.20 Wh.kg^−1^) and electrochemical charge–discharge cycling durability with 88% capacitance retention after 5000 cycles. This work, thus, demonstrates a high potentiality of the Cu@Fe-MOF/NF film electrodes in electrochemical energy-storing devices.

## 1. Introduction

As a result of advancements in technology and human living standards, we are increasingly facing severe environmental and resource issues. In particular, the limited availability of fossil fuels and their unfavorable environmental impact is knocking the scientists to prioritize their research toward green and sustainable energy [1,2]. To address this issue on the future energy crisis under the environmental harmony, researchers are working to develop high-performance energy conversion and energy storage devices—for example, lithium-ion (Li-ion) batteries [3,4,5], supercapacitors [6,7,8], and fuel cells [9,10,11]. Amongst these, supercapacitors (SCs) have gained increasing attention as electrochemical energy storage devices due to their high energy storing capacity, high power density, high electrochemical durability, and environmental friendliness. SCs are, therefore, considered a widely recognized electrochemical energy storage system [6]. The low energy density of the majority of SCs, however, severely limits their wide practicability [12]. Hence, improving energy density without hampering the power density is the primary objective in SCs’ development.

Interestingly, SCs’ properties can be tuned by choosing an appropriate electrode material. However, designing and fabricating electrode materials with specific structures and properties to achieve high-performance SCs is challenging. Various materials, including metal oxides [13], metal chalcogenides [14], conductive polymers [15,16], and carbonous materials [17] have been widely used for SCs.

Metal–Organic Frameworks (MOFs) are considered enormously emerging electrode materials in energy storage devices due to their highly ordered porous channels with abundant redox capacitive metal centers, enabling the storage and free flow of charges between the electrode and electrolyte during charging/discharging cycles. In addition, the flexibility in their synthesis via solution chemistry routes, tunability in the porous structure with high surfaces, and limitless possibilities in the assembly of metal ions and the organic ligands with tunable functionality delimit MOFs as electrochemical energy-storing materials [18,19,20]. So far, several MOFs with diverse structures have been synthesized and utilized in supercapacitor applications. [20,21,22,23,24,25,26,27,28,29,30,31,32]. There are several reliable reviews discussing MOF-based supercapacitor electrode materials and the recent reports focusing on MOFs in supercapacitor applications are discussed in these reviews [21,33,34]. Because of their limitless co-ordination ability with the transition metals, MOFs with transition metal centers such as, Ni, Cu, and Fe are known for being good for electrochemical performance and are commonly utilized [35,36,37,38]. For example, Mei and coworkers prepared a bimetallic Co/Ni MOF, used as a sacrificial template to prepare an accordion-like ternary NiCo_2_O_4_/-NixCo_1_ x(OH)_2_/-NixCo_1_ x-(OH)_2_ composite for supercapacitor application [39]. Moreover, numerous practical methods have been proposed to achieve higher specific capacitance. One common approach is to combine metal–organic frameworks (MOFs) with other functional materials to enhance the overall performance of electrode materials. This can involve improving specific surface areas, increasing electrical conductivity, and enhancing electrolyte compatibility. For instance, a study by Xu and colleagues demonstrated the synthesis of ZIF-67/polypyrrole tube networks, resulting in improved conductivity and a larger specific capacitance (597.6 F g^−1^) compared to ZIF-67 (99.2 F g^−1^) [40]. Another example is the fabrication of the CNT@Mn MOF by Zhang and their team, where the incorporation of carbon nanotubes (CNT) led to a significant enhancement in specific capacitance. This material exhibits both high specific capacitance and excellent cycle performance [41]. Moreover, Tian and co-workers designed novel bimetallic metal–organic framework (MOF) materials with a flower-like nanosheet structure grown on electrospun nanofibers (PPNF@M-Ni MOF, M = Co, Zn, Cu, Fe), exhibiting superior electrochemical performance with high specific capacitance (1096.2 F g^−1^) and excellent rates of performance [42]. These metal centers have various valences and, therefore, can be used as capacitive redox centers. Although various methods have been reported for synthesizing metal–organic frameworks (MOFs), the resulting product is typically in a powdery form, which can suffer from limitations such as lower surface area and limited contact between materials and result in slower charge–discharge rates and higher resistance [43,44,45,46]. In contrast, using direct-grown films or ink-type suspension electrodes offers several benefits in terms of charge–discharge rate and resistance contributions. Ink-type suspensions allow for better electrode morphology and thickness control, resulting in improved charge transport and reduced resistance. Additionally, ink-type suspensions provide higher surface area and enhanced contact between active materials and electrolytes, leading to faster charge–discharge rates and improved electrochemical performance. However, the growth of MOF thin films on conducting substrates for direct utilization in supercapacitors and other electrochemical applications remains challenging.

To address the challenge, a novel Cu@Fe-MOF/NF electrode is prepared via simply drop-casting of the MOFs inks on a 3D porous nickel foam (NF) substrate. The subsequent Cu@Fe-MOF/NF electrode demonstrated a high specific (Cs) capacitance of 562.1 F g^−1^ at 3 A g^−1^, which is twice related with the Fe-MOF/NF (260 F g^−1^) electrode. Moreover, an ASSSC device that uses reduced graphene (rGO) as the anodic (positive) electrode and Cu@Fe-MOF/NF as the cathodic (negative) electrode achieved a high energy density of 44.20 Wh.kg^−1^. The ASSSC device performs securely over an extended period of time, maintaining 88% of capacitance even after 5000 continuous charge/discharge cycles.

## 2. Materials and Methods

### 2.1. Reagents

Reagent grade iron chloride (FeCl_3_·6H_2_O), acetic acid (CH₃COOH), 2-amino terephthalic acid (C_8_H_7_NO_4_), copper nitrate trihydrate (Cu (NO_3_)_2_·3H_2_O), hydrochloric acid (HCl), N, N-dimethylformamide (DMF, C_3_H_7_NO), methanol (CH_3_OH), acetone (C_3_H_6_O), potassium hydroxide (KOH), and Poly(vinyl alcohol (PVA, Mw = 14,000) were acquired from Republic of Korea (Sigma-Aldrich, Seoul, Republic of Korea). A 0.16 cm thick NF substrate (nickel foam) was obtained from the Alantum Corporation (Seoul, Republic of Korea). The NF substrate was cut into 1 × 5 cm^2^ and washed sequentially for 15 min in 2 molar HCl, ethanol, acetone, and deionized water (DI). The cleaned substrates were dried up at room temperature (RT) overnight.

### 2.2. Preparation of MOF Powder

The powder of Cu@Fe-MOF electrode was prepared solvothermally with the reported procedure with a little modification [47,48]. Briefly, 1.5 mmole of FeCl_3_·6H_2_O, 0.75 mmole Cu(NO_3_)_2_·3H_2_O and 2.25 mmole C_8_H_7_NO_4_ were dispersed in 50 mL DMF. Later, 0.50 mL of acetic acid was poured into the above combination. Next, the solution was stirred to get a homogenous mixture and assigned to a Teflon autoclave and heated for 20 h at 150 °C. The Fe-MOFs were prepared using a similar procedure and reacting FeCl_3_·6H_2_O (2.20 mmol), 2.25 mmol 2-amino terephthalic acid, and 0.55 mL acetic acid in 50 mL DMF at 150 °C. Upon completion of the reaction, a precipitate of the MOFs was gathered and washed numerous times with DMF, dried at a room temperature, and utilized for further characterization.

### 2.3. Drop-Cast Coating MOF Thin Films

In an ultrasonic water bath, 5.00 mg MOF bulk powder was dispersed in a 1 mL anhydrous DMF solvent and ultrasonicated for 2 h at a 38.1 kHz frequency. The produced homogenous ink was carefully dripped drop by drop over a conducting NF until it soaked the entire surface of the NF with ink. Each piece of Ni foam electrode can retain approximately 3.06 mg cm^−2^ of the active material. Finally, the drop-cast coated Cu@Fe-MOF/NF film and Fe-MOF/NF electrodes were dry at 50 °C immediately, and the electrode film with the geometrical area of 1 × 1 cm^2^ was used as an active surface while masking the remaining area using Teflon tape (Sigma Aldrich, Seoul, Republic of Korea).

### 2.4. Preparation of PVA-KOH-Based Gel Electrolyte

The preparation of appropriate gel electrolytes is crucial for fabricating an ASSSC device because this is responsible for leakage and, most significantly, for the flexibility of the device. Briefly, 2.5 g of PVA was dissolved in distilled water (20 mL, DW) heated at 80 °C along with continuous stirring. Next, 10 mL KOH aqueous solution (1 M) was poured into the PVA solvent. Then, the above solvent mixture was continuously stirred at a room temperature to form a transparent gelatinous/sticky form. The obtained sticky and transparent gel was utilized as an electrolyte and a separator in the assembly of an ASSSC.

### 2.5. Construction of Asymmetric Supercapacitor Device

To construct an asymmetric supercapacitor device, Cu@Fe-MOF/NF and rGO/NF thin films with an active area around (1 × 1 cm^2^) were employed as cathodic (positive) and anodic (negative) electrode materials in an aqueous 1 M KOH electrolyte. In contrast, anodic and cathodic electrodes were bathed in a PVA-KOH-based gel electrolyte and sandwiched to assemble the ASSSC device. For this, the “electrode ∥ PVA-KOH gel electrolyte ∥ electrode” assembly of the ASSSC was hard-pressed in hydraulic pressure.

### 2.6. Electrochemical Measurements

The electrochemical super capacitive performance of the MOF-based electrodes film was examined using a three-electrode cell assembly in a 1.0 M KOH aqueous electrolyte at a RT, wherein the fabricated MOF/NF thin films (an active area of 1 × 1 cm^2^) were employed as a working electrode, a platinum (pt) and a Hg/HgO electrodes were employed as a counter and a reference electrode, respectively. In addition, cyclic voltammetry (CV), galvanostatic charge–discharge (GCD) curves, and electrochemical impedance spectroscopy (EIS) were used to assess the electrochemical capacitive performance of the electrodes.

The specific capacitance *Cs*(F/g) of electrode was estimated from the non-linear GCD curve applying Equation (1) [49,50,51].

(1) $$Cs=\frac{2\times I\times \int Vdt}{m\times \Delta V}$$

where, *I* is the discharge current density (A g^−1^), Δ*V* is the potential window (*V*), *m* is the mass of active electrode materials (*g*), and Δ*t* is the discharge time (*s*).

### 2.7. Electrochemical Measurement with Two Electrode Asymmetric Cell

The electrochemical characteristics of an asymmetric supercapacitor were analyzed through cyclic voltammetry (CV) and galvanostatic charge–discharge measurements. A positive electrode consisting of Cu@Fe-MOF/NF and a negative electrode composed of reduced graphene oxide (rGO), both with similar charge capacities, were employed for the charge–discharge measurements in a two-electrode asymmetric cell. To ensure stable operation of the asymmetric supercapacitor within a wide potential window, it is crucial to balance the charge capacity (not capacitance) of both electrodes. Based on specific capacitance obtained from a three-electrode cell system, the optimized mass ratio loading of active materials for the asymmetric supercapacitor was determined to be 1:08 for the Cu@Fe-MOF/NF and rGO. The fabricated asymmetric supercapacitor with the optimized mass ratio was then evaluated at various current densities ranging from 3 A·g^−1^ to 7A·g^−1^ to assess the specific energy and specific power. The specific capacitance (C, F·g^−1^), specific energy (E, Wh.kg^−1^), and specific power (P, kW·kg^−1^) were calculated from chronopotentiometry curves using Equations (2)–(4), respectively [52,53,54,55]:

(2) $$Cs=\frac{I\times \Delta t}{m\times \Delta V}$$

(3) $$E=\frac{(\frac{1}{2})Cs\times {\left(\Delta V\right)}^{2}}{3.6}$$

(4) $$P=\frac{E\times 3600}{\Delta t}$$

where *E* is the energy density (Wh.kg^−1^) and *P* is the power density (W.kg^−1^) of ASSC.

All samples were subjected to EIS at open circuit (OCP) potential with an amplitude of 10 mV at a 100 kHz to 100 mHz frequency range.

### 2.8. Characterization of Drop-Casted Thin MOF Films

To investigate the chemical state and structural phase of the MOFs/NF, X-ray photoelectron (XPS) spectroscopy was studied via an Escalab 250Xi spectrometer from ThermoFisher (Waltham, MA, USA). The X-ray diffraction (XRD) pattern was obtained using a Malvern PANalytical X’Pert diffractometer (Malvern, UK) equipped with a Cu Kα radiation source (λ = 15,418 nm) with a scanning speed size of 1°/min. Surface topography and elemental components of the MOF films were obtained using a field-emission scanning electron microscope (FE-SEM) and energy dispersive (EDX) X-ray spectrometer from Oxford 6587 (Oxford, UK). Additionally, transmission electron microscopic (TEM) images, HR-TEM images, and elemental mapping images were obtained using a TalosTMF200X transmission electron microscope (TEM) at 200 kV (Oxford, UK).

## 3. Results and Discussion

### 3.1. MOF Thin Film Formation and Characterizations

In this work, solvothermally synthesized MOF inks were applied through drop-cast on an NF substrate to form mechanically resilient MOF thin film electrodes. Initially, the solvothermal reaction was used to produce the bulk MOF powder followed by ultrasonic treatment in DMF solvent.

**Scheme 1:**
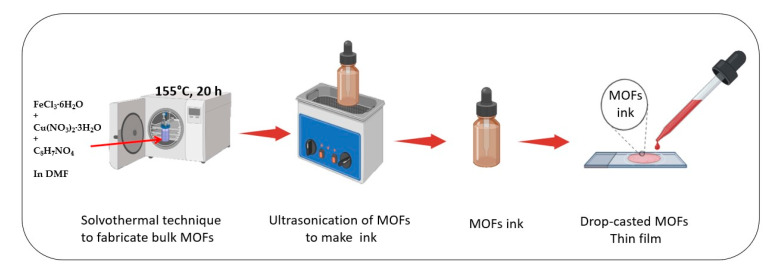
Graphical presentation of MOF thin film fabrication via drop-cast technique on a nickel foam substrate (NF).

**Figure 1:**
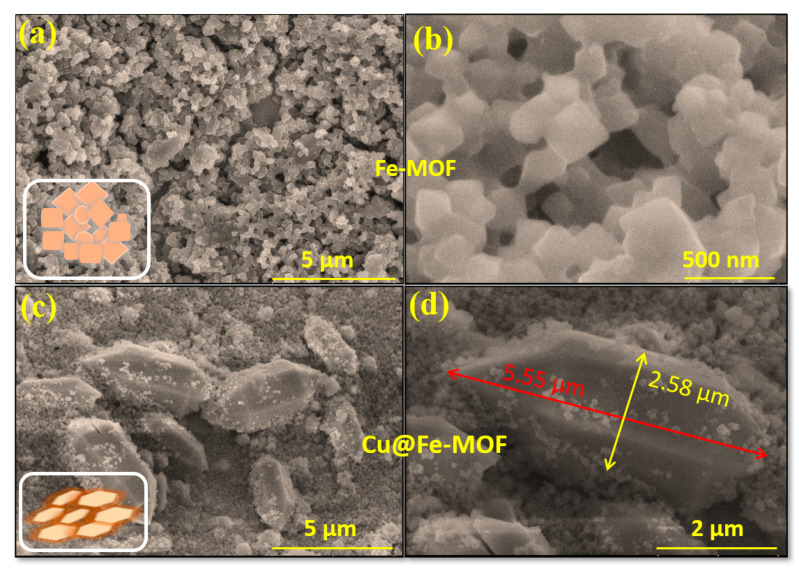
FE-SEM micrographs of the MOF thin film at various magnifications: (**a**,**b**) Fe-MOF/NF, and (**c**,**d**) Cu@Fe-MOF/NF thin films. The inset figure shows the graphical images of the bipyramidal and nanocube structure of the MOFs.

Compared to the traditional methods, this drop-casting route is more efficient for fabricating thin films. In addition, eliminating binders for the film formation makes the approach more versatile and cost-effective and can be adapted easily for various electrode surfaces. In other words, this simple MOF deposition process reduces material waste, making it more cost-effective and efficient. Graphic representation for the MOF thin film formation by the drop-cast route is illustrated in Scheme 1. The surface topography of the drop-casted MOF films analyzed via FE-SEM is presented in Figure 1. Figure 1a,b display SEM images of Fe-MOF/NF electrode film, showing nano-cuboids ranging from 200 to 300 nm. On the contrary, Figure 1c,d illustrate the SEM images of Cu@Fe-MOF/NF electrode film, where the co-ordination of copper and iron metal ions together with the C_8_H_7_NO_4_ ligand resulted in the crystallization of Cu@Fe-MOF in bipyramidal structures composited with the nanoparticles. The bipyramidal structure has a microscale size with a dimension of up to 2.58 × 5.55 µm^2^, as depicted in Figure 1d. These microstructures are surrounded by densely populated nanoscale particles, making abundant electrochemical interfacial regions. In addition, the deposited film has uniformly covered the NF substrate, which is advantageous for achieving good electrochemical performance.

**Figure 2:**
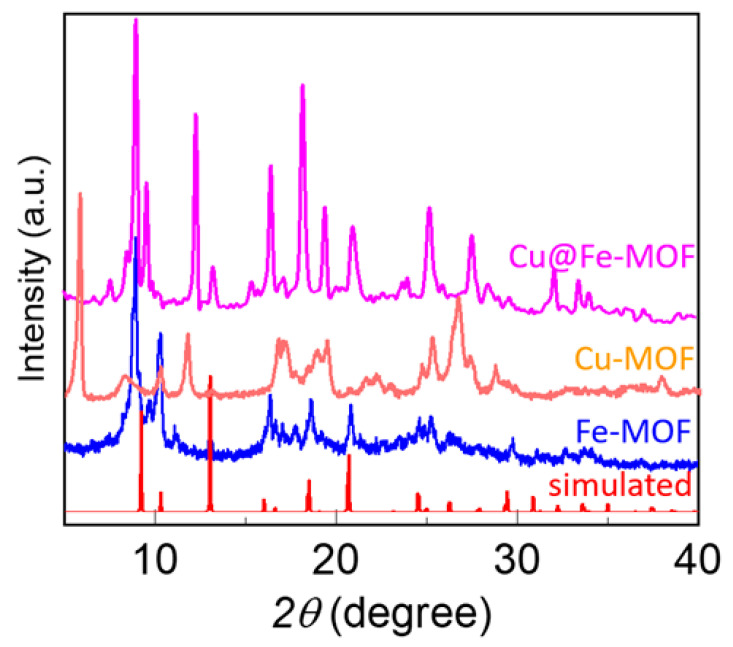
X-ray diffraction patterns of powder MOF.

Due to the prominent XRD peak of the NF substrate, the thin layer of MOF film deposited on an NF substrate made it challenging to access the core MOF peak for analysis using thin film X-ray diffraction (XRD). Therefore, powder samples of the MOFs were compiled from the solvothermal procedure and analyzed, as presented in Figure 2. The XRD diffraction patterns of both MOFs were found to be closely matched with those of the NH_2_-MIL-88B(Fe_2_Ni) MOF, suggesting its crystallization in a P63/MMC space group belonging to the hexagonal crystal structure [47,56]. However, the XRD patterns of the Cu@Fe-MOF electrode shows additional peaks at about 12.4°, 19.5°, and 27.6°. To know the source of these peaks, we analyzed the Cu-MOF powder, which showed different XRD patterns than that of the Fe-MOF and Cu@Fe-MOF electrodes. This suggests that Cu coordinates with the ligand through a different pattern. In addition, the above three unknown peaks observed in the Cu@Fe-MOF patterns seem to come from the Cu-MOF. In addition, Figure S1a,b EDX analysis disclosed that the Cu@Fe-MOF/NF electrode included C, N, O, Fe, and Cu in atomic percentages of 70.30%, 6.34%, 15.23%, and 5.53%, and 2.60%, respectively.

**Figure 3:**
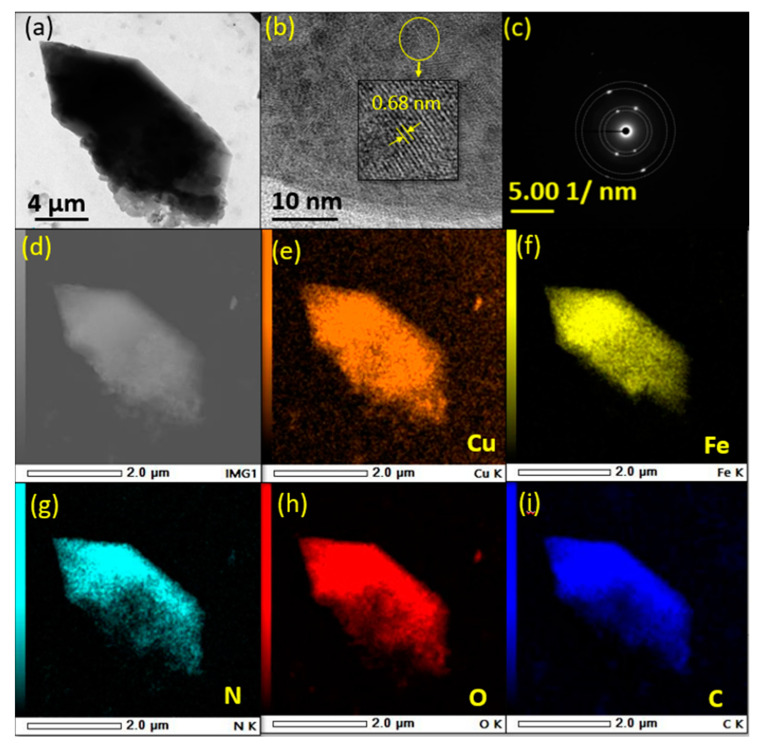
TEM and STEM analysis of Cu@Fe-MOF/NF thin film: (**a**,**b**) TEM image and HR-TEM image; arrows—Inset (**b**): shows *d* spacing of Cu@Fe-MOF/NF), (**c**) SAED pattern of Cu@Fe-MOF/NF, (**d**) HAADF STEM image with uniform distribution of element mapping in (**e**–**i**) which identified orange (Cu), yellow (Fe), green (N), red (O), and blue (C) within the material, providing information on the composition and distribution of elements.

The successful acquisition of TEM images of Cu@Fe-MOF/NF is presented in Figure 3. The thin film of MOFs was immersed in ethanol solvent and sonicated for several minutes to obtain the sample for TEM analysis. As evident in Figure 3a, TEM images of Cu@Fe MOF/NF demonstrated a micro-bipyramidal morphology, which aligns with the SEM results. The HR-TEM picture revealed distinct lattice fringes (Figure 3b). The inset figure shows a zoomed view of the yellow highlighted square part of lattice fringes having the d-spacing of 0.68 nm, which, based on the CIF file of the NH_2_-MIL88B, shows a peak at about 13° corresponding to the (102) lattice plane.

In addition, a selected area electron diffraction (SAED) assessment reveals the polycrystallinity of the Cu@Fe-MOF/NF film in Figure 3c, which is in line with the XRD analysis, as depicted in Figure 2. Furthermore, the high-angle annular dark field scanning transmission (HAADF STEM) electron microscopy image in Figure 3d–i demonstrated the uniform distribution of all the elements in the Cu@Fe-MOF/NF film.

**Figure 4:**
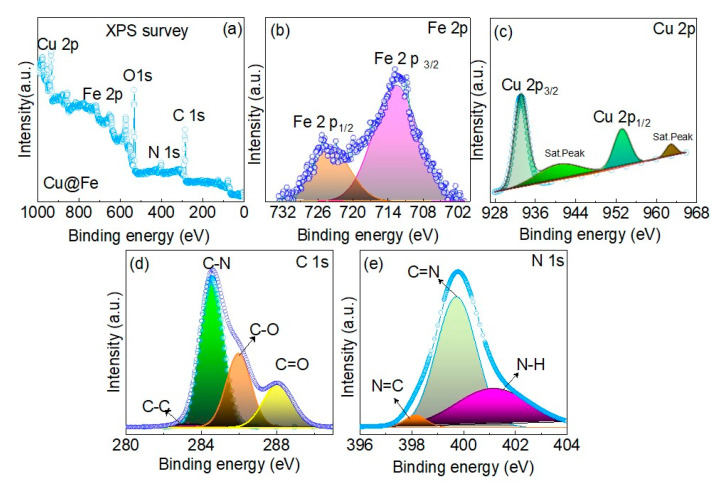
XPS spectra of Cu@Fe-MOF/NF thin film: (**a**) XPS survey spectrum, (**b**) Fe 2p, (**c**) Co2p, (**d**) C 1s, and (**e**) N 1s spectrum.

XPS was used to study the chemical and binding states of elements in Cu@Fe-MOF/NF. The XPS survey in Figure 4a reveals the existence of Cu, Fe, N, O, and C elements. Figure 4b illustrates the high-resolution Fe 2p spectrum centered at 713.57 and 725.01 eV, corresponding to the Fe 2p_3/2_ and Fe 2p_1/2_ orbitals, respectively. This result suggests that the Cu@Fe-MOF/NF electrode contains Fe centers in the 3^+^ oxidation state [56,57]. Similarly, the two prominent doublet centers at 953.28 eV and 933.21 eV observed in Figure 4c correspond to the Cu 2p_1/2_ and Cu 2p_3/2_ orbitals, respectively. A pair of accompanying satellite peaks at 943.71 eV and 962.93 eV was also observed [58]. Furthermore, the C 1s spectra in Figure 4d suggests that the C-N and the C=O bonds associated to the benzene ring of the organic ligand were present. Additionally, the N 1s spectra illustrated in Figure 4e showed the existence of C=N bonding. These results assumed that the amino-terephthalic acid ligand (C8H7NO4) is coordinated with the Cu and Fe ions to form bimetallic Cu@Fe-MOF.

### 3.2. Electrochemical Supercapacitive Performance of the MOF Films

**Figure 5:**
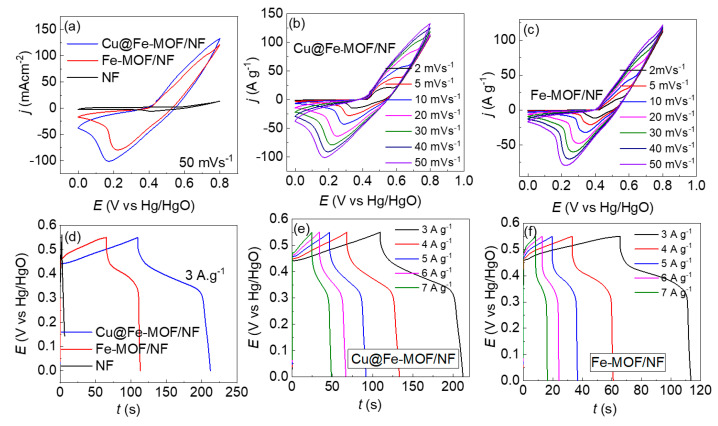
(**a**) CV curve of the MOF film and NF at a constant scan speed of 50 mV s^−1^, (**b**) CV curve of the Cu@Fe-MOF/NF thin films electrodes at various scan rates of 5–50 mV s^−1^, (**c**) CV plots of the Fe-MOF/NF thin films electrodes at various scan rates of 5–50 mV s^−1^, (**d**) GCD curves of the MOF thin films and NF electrodes at a 3 A g^−1^ fixed current density, and (**e**,**f**) GCD curves of Cu@Fe-MOF/NF and Fe-MOF/NF thin films at different current densities of 3–7 A g^−1^, respectively.

The electrochemical performance of the as-fabricated Cu@Fe-MOF/NF and Fe-MOF/NF electrodes was performed using a conventional three-electrode system in an aqueous electrolyte (1.0 M KOH). The cyclic voltammetry (CV) curves presented in Figure 5a depict the performance of the Cu@Fe-MOF/NF and Fe-MOF/NF electrodes at a scan speed of 50 mVs^−1^ in a range of a potential window of 0 V to 0.8 V (vs. Hg/HgO).

This clearly demonstrates the pseudocapacitive behaviors of the MOF thin film samples. In addition, the area under the CV curves of the Cu@Fe-MOF/NF electrode is relatively larger than that of the pristine electrode of Fe−MOF/NF.

**Figure 6:**
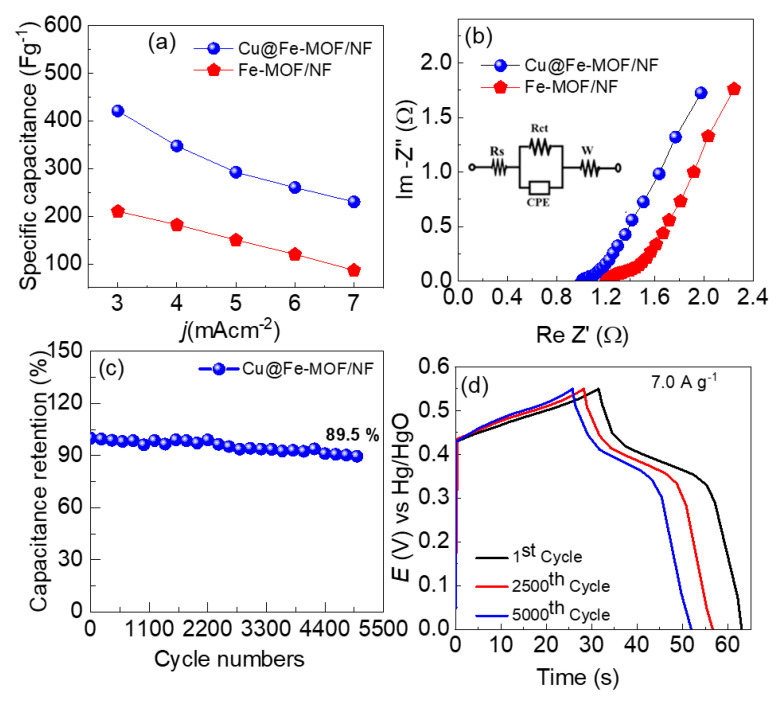
(**a**)The curve of the Cs vs. current density j (A g^−1^) of Cu@Fe-MOF/NF and Fe-MOF/NF, (**b**) Nyquist plots [inset figure: equivalent circuit] of the MOF/NF thin films, (**c**) GCD stability curve for 5000 cycles of Cu@Fe-MOF/NF film, and (**d**) 1st, 2500th, and 5000th GCD cycles of the Cu@Fe-MOF/NF electrode.

This suggests that the dual redox capacitive metal centers in the Cu@Fe-MOF/NF electrode can store more electrical charges. Figure 5b,c demonstrates that the Cu@Fe-MOF/NF electrode and Fe-MOF/NF electrodes deliver the higher electrochemical capacitance with rising the scan rate (2 to 50 mVs^−1^). Note that the shapes of the voltammograms in all the potential scanning rates are similar, suggesting the electrochemical reversibility of the electrode materials. Furthermore, the GCD characteristics of the MOF film-based electrodes were conducted under a constant current density of 3, 4, 5, 6, and 7 A g^−1^ in a potential window range from 0 to 0.55 V vs. reference electrode Hg/HgO (Figure 5d–f). Unlike the symmetric triangular curve in the case of the electric double-layer capacitor (EDLC), the obtained GCD curves of this work shows a non-linear shape, which is the battery-like characteristics, revealing the pseudocapacitive faradaic reaction [59]. This finding is also in line with the CV results. The longer discharge durations of the Cu@Fe-MOF/NF electrode materials show that the capacitance of the Cu@Fe-MOF/NF materials was significantly improved after introducing the Cu-metallic centers to the pristine Fe-MOF/NF. The specific capacitances of the Cu@Fe-MOF/NF and Fe-MOF electrode materials were determined using their GCD curves using the Equation (1), and the results are shown in Figure 6a. The estimated specific capacitance for the Cu@Fe-MOF/NF electrode at a low current density of 3 A g^−1^ is found to be 420 F g^−1^, comparatively higher than that of the Fe-MOF/NF electrode (210 F g^−1^). While the specific capacitances of the Cu@Fe-MOF/NF samples were 347, 292, 260, and 230F g^−1^ at various current densities of 4, 5, 6, and 7 A g^−1^, the Fe-MOF/NF sample showed the lower specific capacitance of 182, 150, 120, and 87 F g^−1^, correspondingly. The superior electrochemical performance of the Cu@Fe-MOF/NF electrode is accredited to the collaborative coordination of the copper (Cu) and iron (Fe) metal ions with the organic ligand, which provides a favorable environment for supercapacitor applications. In addition, the distinct bipyramidal structures making a large number of interfacial regions with the nanoparticles in the Cu@Fe-MOF/NF electrode were favorable for offering the larger active specific surface areas, which ultimately delivered a high charge–discharge rate.

The conductivity, as well as the diffusion behavior of the electrodes, were acquired using the EIS procedure. Figure 6b and Figure S2 illustrate a comparison of the Nyquist plots obtained from the bare NF substrate and from the MOF/NF-based electrodes. The Nyquist plots of these electrodes exhibit series resistance (Rs) of 1.21 ohm for Cu-MOF/NF and 0.988 ohms for the Cu-MOF/NF and Cu@Fe-MOF/NF electrodes, respectively. The Nyquist plots of these electrodes exhibited a half-circle arc corresponding to the charge transfer (Rct) resistance at the high-frequency zone, while a straight line in the low-frequency zone indicates the Warburg diffusion element. The Rct of the Cu@Fe-MOF/NF electrode film is lower (0.97 ohm) than the Fe-MOF/NF electrode (1.17 ohm). The lower Rs and Rct of the Cu@Fe-MOF/NF film electrode is due to the electronic modulation caused by the introduction of more electronegative Cu metal centers to the pristine electrode of Fe-MOF, thereby improving the electrical conductivity of the frameworks.

Additionally, the slope of the Warburg line is also slightly higher in the case of the Cu@Fe-MOF/NF film electrode than in the Fe-MOF/NF electrode. These observations indicate that the Cu@Fe-MOF/NF film electrode exhibits superior electrochemical performance. Owing to the superior performance, the Cu@Fe-MOF/NF electrode was subjected to a long-term electrochemical charge/discharge cycling stability test at up to 5000 cycles at 7 A g^−1^ current density in 1.0 M KOH electrolyte. Notably, Figure 6c indicates that the Cu@Fe-MOF/NF electrode showed robust cycling stability with 89.5% of capacity retention. As evidence, Figure 6d illustrates the 1st, 2500th, and 5000th GCD cycles, which indicate a slight decrease in the storage capacity with the GCD cycles.

**Figure 7:**
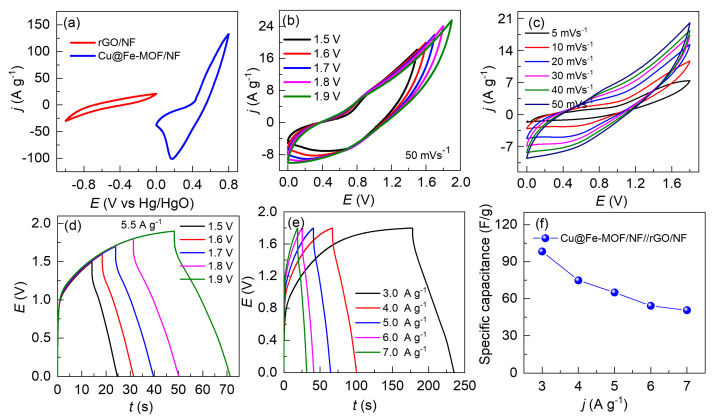
(**a**) CV curves of Cu@Fe-MOF/NF and rGO/NF electrodes at 50 mVs^−1^ scan rate, (**b**) CV curves of the Cu@Fe-MOF/NF//rGO/NF ASSSC device at potential windows of 1.5 to 1.9 V, and (**c**) at diverse scan rates, (**d**) GCD spectrum at potential windows of 1.5 to 1.9 V and (**e**) at 3–7 A g^−1^ current densities, (**f**) specific capacity vs. current density curve of Cu@Fe-MOF/NF and rGO/NF electrodes.

Selecting a suitable potential window is critical for the asymmetric solid-state supercapacitor (ASSSC) device. Therefore, first a Cu@Fe-MOF/NF cathode and an rGO/NF anode were utilized to construct an ASSSC device in 1.0 M KOH aqueous electrolyte. Figure 7a displays the CV plots of the Cu@Fe-MOF/NF and rGO/NF electrodes in a three-electrode assembly at a scan rate speed of 50 mV s^−1^. The rGO/NF electrode functioned between 0 to −1 V, while the Cu@Fe-MOF/NF electrode film was operated with a potential window in the range of 0 to + 0.8 V. The utilization of two distinct electrodes in the ASSSC device with a well-balanced combination can enable a wider range of operating potential, enhancing its overall performance. Further, an ASSSC device has been developed to prevent internal shortening caused by electrolyte leakage, and the device assembled using a Cu@Fe-MOF/NF cathode and a rGO/NF anode sandwiched in a polymer gel electrolyte (Cu@Fe-MOFǁPVA-KOHǁrGO). To define an optimized potential window, the device was cycled at a scan rate of 50 mV s^−1^ while the potential window was gradually changed from 0.0~+1.5 to 0.0~+1.9. The process and results of this optimization are illustrated in Figure 7b. The region under the CV curve spectrum is improved with rising the potentials, as witnessed in Figure 7b. The as-assembled ASSSC device displays good capacitive characteristics by maintaining a quasi-rectangular CV spectrum under the potential window up to +1.8 V.

**Figure 8:**
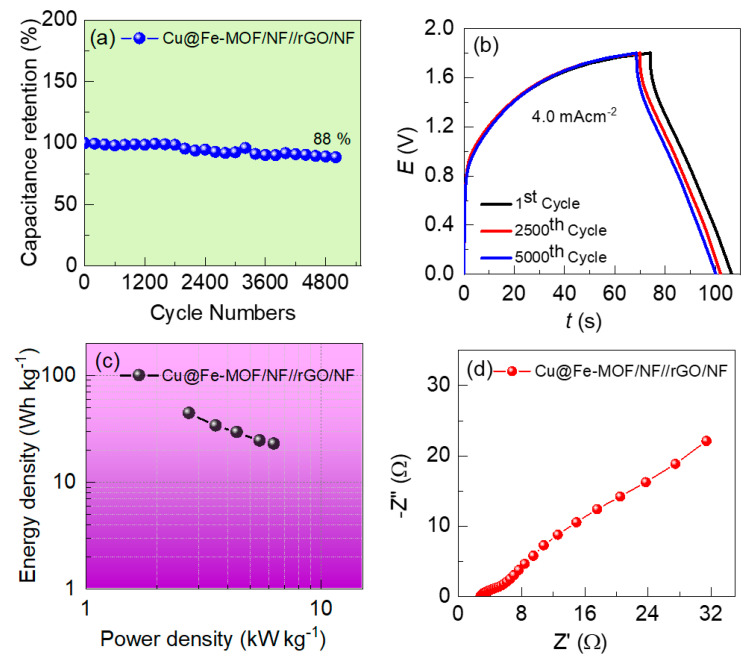
(**a**) Stability graph of Cu@Fe-MOF/NF and rGO/NF for 5500, (**b**) GCD Plots of Cu@Fe−MOF/NF and rGO/NF at 1st, 2500th, and 5000th cycles, (**c**) Ragone plot and (**d**) Nyquist plot of the Cu@Fe-MOF/NF//rGO/NF ASSSC device.

In addition, Figure 7c depicts the complete CV curves of the Cu@Fe-MOFǁPVA-KOHǁrGO device acquired at different sweep speeds varying from 5 to 50 mV s^−1^. All CV curves spectrum in Figure 7c maintain their shapes at different scan speeds, suggesting better capacitive properties of the device. The shape of the CV plots was maintained when the scan speed increased, confirming the outstanding electrochemical reversibility of the Cu@Fe-MOFǁPVA-KOHǁrGO device. Similarly, Figure 7d shows the GCD curves for the Cu@Fe-MOF/PVA-KOH/rGO-based ASSSC device, which reveals the rising charge and discharge times for potentials between +1.5 and +1.9 V at a based device tested at a current density of 3, 4, 5, 6, and 7 A g^−1^ within the 0 to 1.8 V potential window. The GCD curves show the pseudocapacitive charge storage mechanism of the Cu@Fe-MOFǁPVA-KOHǁrGO device. The specific capacitance (Cs) of the Cu@Fe-MOFǁPVA-KOHǁrGO-based device was determined to be 98.23, 74.86, 65.04, 54.20, and 50.64 F g^−1^ at 3, 4, 5, 6, and 7 A g^−1^, respectively, as displayed in Figure 7f. Furthermore, the cycling stability of the ASSSC device is measured for 5000 GCD cycles at a 4.0 A g^−1^, and the capacitance retention of 88% is obtained at the end of 5000 cycles, as shown in Figure 8a. As evidence, the 1st, 2500th, and 5000th GCD cycles are shown in Figure 8b, which reveals a slight decrease in the storing capacity with cycling.

In addition, Figure 8c illustrates the Ragone plot, which presents the power and energy densities at varying current densities. The ASSSC device achieved a highest energy density of 44.20 Wh.kg^−1^ at a power density of 2.743 kW.kg^−1^. Table S1 shows lists of several reported values on asymmetric devices based on MOFs that were published previously. The EIS analysis was conducted to evaluate the charge transfer capability of the Cu@Fe-MOFǁPVA-KOHǁrGO-based device. Figure 8d depicts the Nyquist plot, and an equivalent circuit of the device is shown in the inset of Figure 8d. The results show the Rs = 2.7 Ω and Rct = 6.9 Ω, revealing a high electrical conductivity and excellent electrochemical responses at the electrode/electrolyte interface.

**Figure 9:**
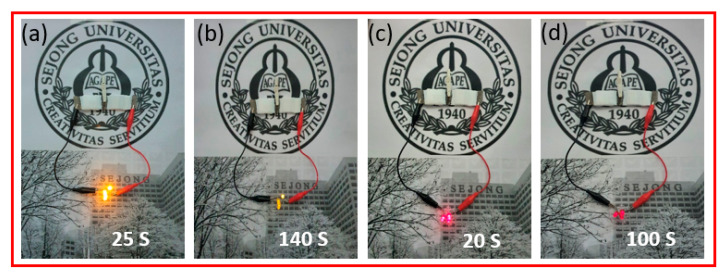
Photographic demonstrations of the ASSSC device by shining (**a**,**b**) yellow and (**c**,**d**) red LEDs for 140 and 100 s, respectively.

To investigate the capability of the ASSSC device, a pair of charged devices were assembled in series and employed for better explanation. At first, the successively connected pair of the ASSSC devices were charged for 30 s and then discharged by connecting to the diverse-colored light-emitting diodes (LEDs) as loads, and were illuminated for around 140 and 100 s, respectively (Figure 9a–d). Note that LEDs require a specific voltage for operation; for example, the yellow LED requires 2.0 V and the red one requires 1.8 V. The initial glorious glow of the LEDs indicates a high-power demonstration and a lengthy discharge affirming the superior energy density of the ASSSC device. Thus, the ASSSC device highlights a good electrical energy-storing capability.

## 4. Conclusions

In this study, Cu@Fe-MOF and Fe-MOF thin films were grown on NF substrates using a drop-cast method. When used for electrochemical supercapacitor applications, the Cu@Fe-MOF/NF electrode displayed relatively excellent electrochemical performance compared to the pristine electrode (Fe-MOF/NF). Interestingly, the Cu@Fe-MOF/NF film electrode achieved a maximum (Cs)specific capacitance of 562.01 Fg^−1^, a two-fold higher figure than that of the pristine Fe-MOF/NF electrode (261 F g^−1^) at a 3 A g^−1^. Remarkably, the Cu@Fe-MOF/NF electrode demonstrated a capacitive retention of 89.5% after 5000 charge/discharge cycles, indicating its superior cycling stability. The improved electrochemical performance of the Cu@Fe-MOF/NF electrode was ascribed to synergistic influence of the copper (Cu) and iron (Fe) metal centers on film morphology, offering larger electrochemically active interfacial regions, and enhanced charge transfer capabilities.

Moreover, the ASSSC devices achieved an utmost specific capacitance (Cs) of 37 F g^−1^ at 3 A g^−1^. Most importantly, the devices achieved a maximum specific energy and the specific power of 44.20 Wh.kg^−1^ and 2.743 kW kg^−1^, respectively. In addition, the ASSSC devices showed a capacitance retention of 88% at the end of 5000 charge/discharge cycles, indicating the practicability of the device. Furthermore, the practical feasibility of the ASSSC device was demonstrated by illuminating an LED bulb for 140 s, operating a series combination of two ASSSC devices. The excellent electrochemical performance and cycling stability suggests that drop-casting of the Cu@Fe-MOF/NF film electrode has significant potentiality as a cathode material for future energy storage.

## Data Availability

The data presented in this study are available in this article and Supplementary Materials.

## Supplementary Materials

The following supporting information can be downloaded at: https://www.mdpi.com/article/10.3390/nano13101587/s1, Figure S1: EDS analysis of Cu@Fe-MOF/NF thin film. (a) atomic percentage,(b) corresponding spectrum of EDS.; Figure S2: Capacitive contribution from the nickel foam current collector. (a) Cyclic voltammetry, (b) GCD curve; Table S1: Comparison on the electrochemical performances of the representative materials tested in a three-electrode system.
