# Supplementary material for: Cu@Fe-Redox Capacitive-Based Metal–Organic Framework Film for a High-Performance Supercapacitor Electrode
Source: Nanomaterials (Basel). 2023 May 9;13(10):1587. doi: 10.3390/nano13101587 (PMC10222974; doi:10.3390/nano13101587)
Supplement: Supplementary file 1 [file nanomaterials-13-01587-s001.zip › nanomaterials-2361439-supplementary.pdf]

Supporting Information

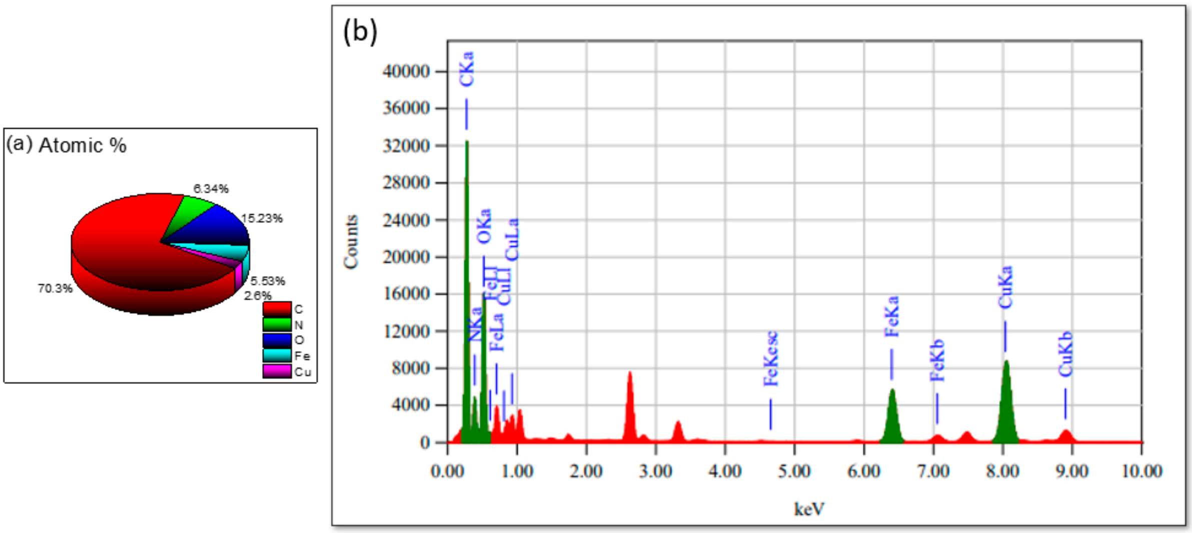

Figure S1. EDS analysis of Cu@Fe-MOF/NF thin film. (a) atomic percentage,(b) corresponding spectrum of EDS.

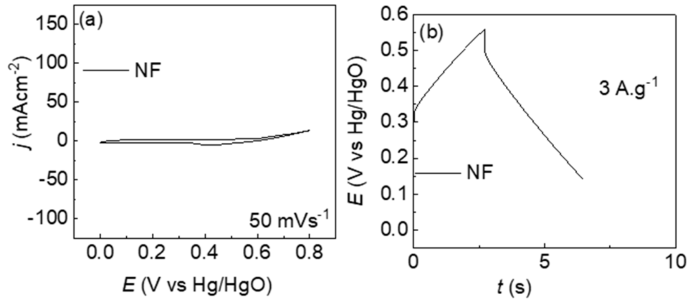

Figure S2. Capacitive contribution from the nickel foam current collector. (a) Cyclic voltammetry, (b) GCD curve.

Table S1. Comparison on the electrochemical performances of the representative materials tested in a three-electrode system.

Sr. No	Materials	Capacitance (F/g)	Current density ($mAcm^{-2}$)	Electrolyte (M)	References
	Cu@Fe-MOF/NF	562	3	1 M KOH	This work
	Fe-MOF/NF	260	3	1 M KOH	This work
1	Ni_1Co_2MOF	445	2	1 M KOH	[1]
2	Co_3O4/CAs composite	298.8	0.5	2 M KOH	[2]
3	NHCSF-3	253	1	6 M KOH	[3]
4	ZCCA	241.6	0.5		[4]
5	SSKOH800	356.23	0.1	2 M Li_2SO_4	[5]
6	C-IPN3	239.1	0.5	6 M KOH	[6]
7	Carbon-ZS	285.8	0.1	6M KOH	[7]
8	NSPC	224.3	1	6M KOH	[8]
9	HNSCs-x	230	0.5	6M KOH	[9]
10	NHPC	234	0.5	6M KOH	[10]

References

1. Liang, Y.; Yao, W.; Duan, J.; Chu, M.; Sun, S.; Li, X. Nickel Cobalt Bimetallic Metal-Organic Frameworks with a Layer-and-Channel Structure for High-Performance Supercapacitors. *J. Energy Storage* **2021**, *33*, 1–11, doi:10.1016/j.est.2020.102149.

2. Wang, M.X.; Zhang, J.; Fan, H.L.; Liu, B.X.; Yi, X. Bin; Wang, J.Q. ZIF-67 Derived Co3O4/Carbon Aerogel Composite for Supercapacitor Electrodes. *New J. Chem.* **2019**, *43*, 5666–5669, doi:10.1039/c8nj05958f.

3. Li, Z.; Mi, H.; Liu, L.; Bai, Z.; Zhang, J.; Zhang, Q.; Qiu, J. Nano-Sized ZIF-8 Anchored Polyelectrolyte-Decorated Silica for Nitrogen-Rich Hollow Carbon Shell Frameworks toward Alkaline and Neutral Supercapacitors. *Carbon N. Y.* **2018**, *136*, 176–186, doi:10.1016/j.carbon.2018.04.075.

4. Wang, M.; Zhang, J.; Yi, X.; Zhao, X.; Liu, B.; Liu, X. Nitrogen-Doped Hierarchical Porous Carbon Derived from ZIF-8 Supported on Carbon Aerogels with Advanced Performance for Supercapacitor. *Appl. Surf. Sci.* **2020**, *507*, 145166, doi:10.1016/j.apsusc.2019.145166.

5. Hu, J.; Wang, Z.; Si, Y.; Hong, C.; Zhao, C.; Xing, Y.; Ling, W.; Wang, Y.; Feng, L.; Feng, W. Construction of Shrimp Shell (SS) Waste-Based Carbon Electrode-Gel Polymer Electrolyte (GPE) System for Flexible Symmetric Supercapacitors. *J. Mater. Chem. A* **2022**, *11*, 878–890, doi:10.1039/d2ta07795g.

6. Zhang, L.C.; He, Z.H.; Hou, J.F.; Kong, L. Bin Influence of Subnanoporous Carbon with a Customizable Pore Structure on Aqueous Supercapacitors. *ACS Appl. Energy Mater.* **2022**, *5*, 7081–7090, doi:10.1021/acsaem.2c00699.

7. Zhong, S.; Zhan, C.; Cao, D. Zeolitic Imidazolate Framework-Derived Nitrogen-Doped

Porous Carbons as High Performance Supercapacitor Electrode Materials. *Carbon N. Y.* **2015**, *85*, 51–59, doi:10.1016/j.carbon.2014.12.064.

8. Xiao, J.; Wang, Y.; Zhang, T.C.; Yuan, S. N,S-Containing Polycondensate-Derived Porous Carbon Materials for Superior CO2 Adsorption and Supercapacitor. *Appl. Surf. Sci.* **2021**, *562*, 150128, doi:10.1016/j.apsusc.2021.150128.

9. Ke, C.C.; Zhang, N.; Liu, F.; Yu, Q.; Wang, F.Y.; Liu, L.; Zhang, R.L.; Liu, X.; Zeng, R.C. Deflated Balloon-like Nitrogen-Rich Sulfur-Containing Hierarchical Porous Carbons for High-Rate Supercapacitors. *Appl. Surf. Sci.* **2019**, *484*, 716–725, doi:10.1016/j.apsusc.2019.04.132.

10. Zeng, R.; Tang, X.; Huang, B.; Yuan, K.; Chen, Y. Nitrogen-Doped Hierarchically Porous Carbon Materials with Enhanced Performance for Supercapacitor. *ChemElectroChem* **2018**, *5*, 515–522, doi:10.1002/celc.201701021.
